# Effects of body weight and posture on pulmonary functions in asthmatic children

**DOI:** 10.4314/ahs.v20i4.31

**Published:** 2020-12

**Authors:** Ghobrial Emad Emil, El Baz Mohamed Saad, Abdel Fattah Mohammed, Haroun Manar Mohamed

**Affiliations:** 1 Department of Pediatrics, Kasr Al Ainy School of Medicine, Cairo University, Cairo; 2 Department of Pediatrics, Ministry of Health, Cairo

**Keywords:** Body weight, posture, asthmatic children

## Abstract

**Background:**

Asthma is one of the most common chronic illnesses in the world. Pulmonary function tests are important tools in monitoring of asthmatic patients. There is need for investigating if spirometric indices were affected by body weight or posture or not.

**Objectives:**

The aim of this study was to evaluate the spirometric measurements in standing and sitting positions in a group of Egyptian asthmatic children with different body weights.

**Methods:**

Sixty patients were included. They were stable asthmatics and were following up in the allergy clinic. Spirometry was conducted at pulmonary functions laboratory of Pediatric Allergy and Chest Unit of New Children's University Hospital, Cairo. The one-way analysis of variance was used to test the differences between groups. The Duncan multiple comparison test was used to test the significant differences between each pair of groups.

**Results:**

The study found that sitting FEV1/FVC is significantly lower in overweight/obese asthmatic children compared to normal weight asthmatic children (p value=0.046).

**Conclusion:**

There was no effect of weight on standing spirometric data. Weight showed significant negative correlation with asthma control level. We concluded that in overweight/obese asthmatic children, spirometric position might affect the results.

## Introduction

Asthma is a heterogeneous disease, usually characterized by chronic airway inflammation. It is defined by the history of respiratory symptoms such as wheeze, shortness of breath, chest tightness, and cough that vary over time and in intensity, together with variable expiratory airflow limitation^1^. Asthma severity can be assessed when the patient has been on regular controller treatment for several months^2^.

The simultaneous increment in the prevalence of obesity and allergic diseases suggests a possible link between them. Obesity is related more strongly to non-atopic than atopic asthma, and the mechanisms of asthma in obese patients could involve mechanical effects of obesity on lung function, adipokines-mediated inflammation, environmental factors and co-morbidities^3^.

In obese individuals, sitting position might interfere with descent of diaphragm and it is possible that this effect will be reduced by standing position^4^.

Pulmonary function tests (PFTS) are an important tool in the investigation and monitoring of patients with respiratory pathology. They provide important information relating to the large and small airways and the pulmonary parenchyma. Spirometry is the most frequently used measurement of lung function and is a measurement of volume against time. It is a simple and quick procedure to perform. Measurements that are made include: Forced expiratory volume in one second (FEV1), forced vital capacity (FVC) and the ratio of the two volumes (FEV1/FVC). Calculation of FEV1/FVC allows the identification of obstructive or restrictive ventilatory defects^5^.

The FEV1/FVC ratio is the most sensitive and specific indicator for identifying airway obstruction. These parameters are compared with reference values. References values have been published for different countries^6^.

Pulmonary functions should be assessed at diagnosis or start of treatment; after 3–6 months of controller treatment to assess the patient's personal best forced expiratory volume in 1 second (FEV1) and periodically thereafter^1^.

American Thoracic Society (ATS) and European Respiratory Society (ERS) recommended performing spirometry in sitting position^7^.

Children have a dynamic developmental phase during which lung volume size and airway size change with increasing age. Spirometry parameters are influenced by weight, height, age, sex, environmental factors, ethnicity, prematurity, patient cooperation and effort and technical factors^8^. Solid information on the effects of position and body weight on forced expiratory manoeuvres in children is still lacking. Thus there is need for investigating if spirometric indices (forced expiratory volume in first second [FEV1], forced vital capacity [FEV], forced expiratory flow after 50% of FVC [FEF 50] and peak expiratory flow [PEF]) were affected by body weight or testing posture or not^9^.

The aim of this study was to evaluate the spirometric measurements in standing and sitting positions in a group of Egyptian asthmatic children with different body mass index (BMI) [(normal weight, overweight and obese].

## Methods

This cross-sectional study was conducted at University Children's' Hospital, Faculty of Medicine, Cairo University, Egypt. Sixty patients were included. They were stable asthmatics diagnosed according to Global Initiative for Asthma guidelines (GINA, 2015)^1^ and were following-up in the chest allergy clinic of the hospital. The study was conducted from August 2015 to January 2016. Sixty consecutive patients regularly following in the chest allergy clinic of Children's' Hospital, Cairo University were assigned to participate in this study. This clinic is a specialized clinic in our tertiary hospital, which received patients referred from the general out-patients' clinics and from outside the hospital.

Sample size calculation was performed using Power and Sample Size Calculator program version 3.0.43. It was based on the following inputs: Power of 80 %, Type I error of 0.05, and within-group standard deviation of 0.2.

They were divided into 3 subgroups based on their body mass index (BMI = weight in kilograms divided by squared height in meters) using the international cutoff points for overweight and obese children published by Cole et al., (2000)^10^; subgroup 1: included asthmatic children with normal body weight; subgroup 2: included overweight asthmatic children and subgroup 3: included obese asthmatic children.

Patients aged from 6 years to 16 years with a diagnosis of asthma (according to GINA guidelines), not receiving bronchodilators before study enrollment were included in the study. Underweight children (weight below 5th percentile for age) and acute asthmatic children were excluded from the study.

Both underweight and children with exacerbation may have concomitant problems that may affect their ability to perform a reliable and reproducible test, as underweight children may have malnutrition that may affect their ability to perform the forced spirometric maneuver, whereas patients with acute exacerbation may have concomitant upper or lower respiratory infections and also they are usually put on inhalation therapy with inhaled bronchodilator that will alter the spirometric data.

The aim and nature of the study was explained for each parent before inclusion and approval to participate in the study is mandatory. An informed written consent was obtained from parents / surrogates before enrollment. The study design conformed to the requirements of Revised Helsinki Declaration of Bioethics (2013). The study protocol was approved by the Scientific Research Committee of Pediatrics Department, Faculty of Medicine, Cairo University.

For all children, the following were recorded; history taking [including age, gender, residence, history of wheezes, cough and shortness of breath, seasonal variation of symptoms, nocturnal symptoms, previous history of similar attacks, history of other atopic manifestations and family history of atopy], physical examination [including general examination (especially heart rate, respiratory rate, and temperature), chest examination: (inspection for shape of chest, respiratory movements and trachea, palpation for tenderness, trachea and chest movement), and auscultation for air entry, type of breathing and rhonchi)].

Assessment of control of asthma in our study depends on history from the parents about number and severity of the attacks and on pulmonary function tests, using GINA guidelines 1; being either controlled, partly controlled or uncontrolled asthmatics.

Body mass index (BMI) was calculated (in kg/m^2^) for each participant. Accordingly, he/she was allocated to the appropriate subgroup.

Spirometry was conducted at the pulmonary functions laboratory of Pediatric Allergy and Chest Unit of New Children's University Hospital using a pediatric spirometer (CareFusion Master Screen Paed®, Germany) according to American Thoracic Society (ATS) recommendations and reference values. A minimum of three forced expiratory maneuvers were performed at each session and the test result with best scores was included in the data analysis. Patients are asked to take a maximal inspiration and then to forcefully expel air for as long and as quickly as possible. Forced vital capacity (FVC), forced expiratory volume in 1 second (FEV1), forced expiratory flow at 25 to 75% of vital capacity (FEF25-75) and peak flow rate (PFR) were measured and the FEV1/FVC ratio was calculated. The spirometry maneuvers were performed in random order in sitting and standing positions for each child with an interval of 15 minutes between the two positions.

Changes of spirometric values in sitting and standing positions were calculated as follows: Percentage of change = [(first position - second position) / second position] × 100. This randomization of the order of tested positions aimed to reduce statistical bias of the results.

## Statistical analysis

The SPSS software (Statistical Package for the Social Sciences, version 19.0, Inc, Chicago, Ill, USA) and Microsoft Excel software program were used to tabulate the results and represent them graphically. Quantitative variables were expressed as mean and standard deviation. Qualitative variables were expressed as count and percentage. The one-way analysis of variance was used to test the differences between groups. The Duncan multiple comparison test was used to test the significant differences between each pair of groups. The chi-square test was used to compare the distributions between groups. The Pearson correlation coefficient test used to test the significant correlations between the quantitative parameters within each group. A P value less than 0.05 is considered significant.

## Results

Our study included 60 asthmatic children, 30 of them were males and 30 were females. Mean age of our patients was 9.70± 0.26 years, their mean weight was 34.75 ± 12.48 kg, their mean height was 133.60± 12.76 and their mean BMI was 19.06± 4.12 kg/m^2^. Patients were classified according to weight into 3 subgroups; subgroup 1: included asthmatic children with normal body weight [39 patients (65%)], subgroup 2: included overweight asthmatic children [14 patients (23.33%)] andubgroup 3: included obese asthmatic children [7 patients (11.67%)].

Twenty-seven patients (45%) were well controlled, 25 patients (41.67%) were partially controlled and 8 patients (13.33%) were not controlled.

Table (1) shows the effect of position on % predicted spirometric data of all cases. There was no significant effect of position on spirometric data of all asthmatic children.

**Table 1:** Effect of position on % predicted spirometric data of all cases

	Sitting position	Standing position	95% CI	P-value
**FEV1**	90.84 ± 28.19	91.04 ± 27.06	-10.20, 9.79	0.968
**FVC**	85.97 ± 29.05	87.50 ± 29.90	-12.19, 9.12	0.776
**FEV1/FVC**	106.77 ± 9.24	105.78 ± 11.21	-2.73, 4.70	0.599
**FEF25**	88.03 ± 36.53	90.96 ± 39.29	-16.65, 10.79	0.673
**FEF50**	82.46 ± 28.67	84.38 ± 31.82	-12.88, 9.03	0.728
**FEF75**	79.96 ± 23.13	80.73 ± 26.02	-9.67, 8.13	0.864
**PEF**	76.71 ± 24.22	78.80 ± 26.42	-11.26, 7.07	0.652

FEV_1_: forced expiratory volume in 1 second

FEV: forced expiratory volume

FVC: forced vital capacity

PEF: peak expiratory flow

FEF: Forced expiratory flow

PFR: peak flow rate

95% CI= Confidence intervals at 95% level

Table (2) shows the effect of position on % predicted spirometric data of normal weight children (39 patients) and overweight/obese children (21 patients). There was no significant effect of position on spirometric data of normal weight asthmatic children. There was also no significant effect of position on spirometric data of overweight/obese asthmatic children.

**Table 2:** Effect of position on % predicted spirometric data of normal weight children (n=39) and overweight/obese children (n=21)

		Sitting position	Standing position	95% CI	P-value
**Normal weight**	**FEV1**	90.8 ± 33.2	90.1 ± 32.2	-14.05, 15.43	0.926
	**FVC**	84.2 ± 33.4	85.2 ± 34.6	-16.39, 14.30	0.892
	**FEV1/FVC**	108.71 ± 7.77	107.11±8.69	-2.12, 5.32	0.395
	**FEF25**	89.2 ± 36.2	93.7 ± 40.4	-21.82,12.78	0.604
	**FEF50**	83.9 ± 32.2 -	87.9 ± 33.8	18.91,10.89	0.594
	**FEF75**	80.6 ± 27.3	82.4 ± 25.4	-13.75,10.07	0.759
	**PEF**	78.1 ± 28.5	80.6 ± 28.0	-15.17, 10.32	0.706
**Overweight/** **Obese**	**FEV1**	91.0 ± 16.0	92.8 ± 13.7	-11.15, 7.41	0.686
	**FVC**	89.3 ± 18.9	91.7 ± 18.1	-13.98, 9.11	0.672
	**FEV1/FVC**	103.2 ± 10.8	103.3 ± 14.7	-8.22, 7.94	0.971
	**FEF25**	85.8 ± 37.9	85.8 ± 37.6	-23.5, 23.6	0.998
	**FEF50**	79.8 ± 21.1	77.9 ± 27.2	-13.29, 17.18	0.798
	**FEF75**	78.9 ± 12.6	77.6 ± 27.4	-12.28, 14.70	0.856
	**PEF**	74.1 ± 13.2	75.6 ± 23.5	-13.47, 10.53	0.804

FEV_1_: forced expiratory volume in 1 second

FEV: forced expiratory volume

FVC: forced vital capacity

PEF: peak expiratory flow

FEF: Forced expiratory flow

PFR: peak flow rate

95% CI= Confidence intervals at 95% level

Table (3) shows the effect of weight on sitting and standing % predicted spirometric values. Sitting FEV1/FVC was significantly lower in overweight/obese group compared to normal weight group (p value=0.046).

**Table 3:** Effect of weight on sitting and standing % predicted spirometric values

		Normal weight (n=39)	Overweight/obese (n=21)	95% CI	*P*- value
**Sitting**	**FEV1**	90.8 ± 33.2	91.0 ± 16.0	-12.90, 12.54	0.977
	**FVC**	84.2 ± 33.4	89.3 ± 18.9	-18.58, 8.44	0.455
	**FEV1/FVC**	108.71 ± 7.77	103.2 ± 10.8	0.12, 10.97	0.046*
	**FEF25**	89.2 ± 36.2	85.8 ± 37.9	-17.1, 23.8	0.741
	**FEF50**	83.9 ± 32.2	79.8 ± 21.1	-9.83, 17.89	0.562
	**FEF75**	80.6 ± 27.3	78.9 ± 12.6	-8.65, 12.05	0.744
	**PEF**	78.1 ± 28.5	74.1 ± 13.2	-6.77, 14.85	0.457
**Standing**	**FEV1**	90.1 ± 32.2	92.8 ± 13.7	-14.68, 9.19	0.647
	**FVC**	85.2 ± 34.6	91.7 ± 18.1	-20.10, 7.18	0.347
	**FEV1/FVC**	107.11 ± 8.69	103.3 ± 14.7	-3.39, 10.99	0.288
	**FEF25**	93.7 ± 40.4	85.8 ± 37.6	-13.2, 29.0	0.453
	**FEF50**	87.9 ± 33.8	77.9 ± 27.2	-6.18, 26.14	0.220
	**FEF75**	82.4 ± 25.4	77.6 ± 27.4	-9.90, 19.40	0.516
	**PEF**	80.6 ± 28.0	75.6 ± 23.5	-8.71, 18.70	0.467

FEV1: forced expiratory volume in 1 second FEV: forced expiratory volume FVC: forced vital capacity PEF: peak expiratory flow

FEF: Forced expiratory flow PFR: peak flow rate 95% CI= Confidence intervals at 95% level.

However, there was no effect of weight on standing spirometric data of asthmatic children.

Correlations between level of asthma control and other variables revealed that weight has significant negative correlation with asthma control level; as weight increases, asthma control worsens (r = -0.262, -0.385, respectively, p= 0.043, 0.002, respectively). However, there were no significant correlations between level of asthma control and age, height and BMI) (table 4).

**Table 4:** Correlations between level of asthma control and other variables (n=60)

	Variables	Results	
**Asthma** **control**	**Age (yrs)**	**r-**	0.056
		**p-value**	0.671
	**Weight (kg)**	**r-**	-0.262
		**p-value**	0.043*
	**Height (cm)**	**r-**	-0.173
		**p-value**	0.187
	**Body mass index** **(BMI)**	**r-**	-0.180
		**p-value**	0.168

## Discussion

The aim of this study was to evaluate the spirometric measurements in standing and sitting positions in a group of Egyptian asthmatic children with different body mass index (BMI). The present study hypothesized that pediatric spirometric values would be affected by the body weight as well as the position of the child during test conduction.

Asthma is one of the most common chronic illnesses in the world, and reports in the Middle East suggest that the prevalence of asthma is increasing. Poor knowledge, fear of the use of new drugs, and the lack of awareness of the importance of disease control are common among primary care physicians who care for asthma patients. In addition to these important factors, there are other attributes to the magnitude of disease burdens such as socioeconomic status, number of siblings, knowledge of caregivers, and income^11^.

Pulmonary function tests (PFTS) are important tools in the investigation and monitoring of patients with respiratory pathology. Spirometry is the most frequently used measurement of lung function and is a measurement of volume against time. It is a simple and quick procedure to perform^5^.

The present study showed that there were no significant differences between standing and sitting positions on spirometric data of all asthmatic children.

Similarly, Razi and Moosavi (2007)^9^ conducted a study on adult obese asthmatics and compared their spirometric values to normal weight asthmatics, and concluded that spirometric values in obese asthmatic patients with BMI ≥30 were not affected by the standing or sitting positions.

On the contrary, Lalloo et al., (1991)^12^ examined the effect of the standing versus the sitting position on spirometric indices in 94 healthy non-obese adult subjects On average, all the spirometric indices examined, except the peak expiratory flow rate, were higher in the standing compared to the sitting position although the change was not significant except for FEV1 in women.

In the current study, sitting FEV1/FVC was significantly lower in overweight/obese asthmatic children compared to normal weight asthmatic children. The lower expired volumes measured in the sitting position were probably due to the overweight/obese children are taking slightly smaller inspirations in sitting posture than in the standing position.

These results appear to be different from the findings of Berntsen et al., (2011)^4^, done for children and adolescents (23 overweight and 92 obese) children (7–17 years old), performed forced expiratory flow-volume manoeuvres in sitting and standing posture in random order, and concluded that FEV1, FVC and FEF50 were significantly higher in sitting compared with standing posture. FEV1/FVC and peak expiratory flow were not significantly different measured in sitting and standing posture. However, there were significant design differences between the two works as Berntsen and his colleagues examined overweight and obese children where only 15% of them were asthmatics, and the rest were not having bronchial asthma, and they did not include normal weight children.

Razi and Moosavi (2007)^9^ included 49 obese asthmatic patients with mean age of 42.63 years and BMI of 36.06 kg/m^2^, and 51 control obese normal that spirometric values in obese asthmatic patients with BMI ≥30 were not affected by the standing and sitting positions.

The present study declared that weight was significantly inversely correlated with asthma control level. This conclusion was supported by Wang and Pan (2016) 13 who investigated the effects of obesity on response to therapy and pulmonary function in children with asthma. A total of 129 children with asthma; normal weight group (n=64) and obese group (n=65) and 68 healthy children (control group). They found that there were significant differences in the indices of pulmonary function between the three groups before treatment (P<0.01); the healthy control group had the best values of pulmonary function, while the obese group had the worst values. After 1 year of treatment, the normal weight group showed significantly more improvements in FEV1% and FVC% than the obese group (P<0.01). The normal weight group had a significantly better asthma control status than the obese group (P<0.01).

Spathopoulos et al., (2009)^14^ aimed to investigate the effect of obesity on pulmonary function tests and secondary the possible link of obesity with atopy and asthma in a large cohort of children (2,715 children aged 6–11 years). Six hundred fifty seven children with BMI > 85th percentile (357 overweight, 300 obese) and a group of 196 normal weight children underwent spirometry. The % expected FVC, FEV1, FEF25-75, and FEV1/FVC were significantly reduced in overweight or obese children compared to children with normal weight (P = 0.007, P < 0.001, P < 0.001, and P < 0.001, respectively).

On the other hand, Andrade et al., (2013)^15^ investigated obesity and asthma by comparing gender, age, initial classification of asthma, clinical control, basal forced FEV1and FEF25-75 with rates of BMI in asthmatic adolescents. The study involved 120 asthmatics patients (1.9 male: 1 female) with a mean age of 14.1 years (9 to 20.1 years of age). They found no significant correlations were found between BMI and FEV1 and between BMI and FEF25-75. They reported that no significant correlation was found between overweight/obesity and asthma using clinical, anthropometric, and spirometric parameters.

The current small study was one of the few works addressing the issue of position and weight on spirometric values of asthmatic children. However, it had its share of limitations; for example, the small sample size, the lack of follow-up, and the small number of children in the obese subgroup. However, authors believe that this might serve as a preliminary report upon which further studies on a larger scale can be conducted in the future.

We did not add control group of normal children as this will make the aim of the work is to study the effects of asthma on pulmonary function tests (PFTS) and this was well-studied before in the literature. We are concerned with the effects of overweight in asthmatic children, on PFT, which is not investigated enough in the literature.

## Conclusion

There is no significant difference between standing and sitting positions on spirometric data of asthmatic children. Sitting FEV1/FVC is significantly lower in overweight/obese asthmatic children compared to normal weight asthmatic children. Weight significantly inversely correlated with asthma control level.

The study recommends conduction of further studies on the effect of posture and overweight and obesity on spirometric results of asthmatic children on a larger number of cases.
